# Blood Lactate’s Relationship with Step Kinematic Asymmetry, Stance-Phase Biomechanics, and Spring–Mass Model Variables at the Transition from Curve to Straight Sprinting in the 200 m Dash: A Cross-Sectional Study

**DOI:** 10.3390/jfmk11030335

**Published:** 2026-08-26

**Authors:** Efthymios Kyprianou, Ploutarchos Saraslanidis, George Tsalis, Vassilios Panoutsakopoulos

**Affiliations:** 1 School of Sport and Exercise Sciences, Liverpool John Moores University, Liverpool L3 3AF, UK; 2 School of Physical Education and Sport Science at Thessaloniki, Aristotle University of Thessaloniki, 54124 Thessaloniki, Greece; 3 School of Physical Education and Sport Science at Serres, Aristotle University of Thessaloniki, 62110 Agios Ioannis, Greece; 4 Biomechanics Laboratory, School of Physical Education and Sport Science at Thessaloniki, Aristotle University of Thessaloniki, 54124 Thessaloniki, Greece

**Keywords:** track and field, sport performance, speed, biomechanical analysis, spring–mass model, stiffness, angular kinematics, work physiology, neuromuscular system, fatigue

## Abstract

**Background**: Curve sprinting in the 200 m dash entails distinct biomechanical requirements under strenuous anaerobic conditions that differ from sprinting on the straightway. This study aimed to examine possible relationships between anaerobic biomarkers (specifically, peak post-test blood lactate concentration; BLa) and step kinematic asymmetry, stance-phase biomechanics, and spring–mass model (SMM) variables at the curve-to-straight transition during a 200 m sprint. **Methods**: Sixteen adult male club- and national-level sprinters performed a maximal 200 m sprint test in this exploratory cross-sectional study. Kinematical data were recorded for the first step immediately after the geometric end of the curve, and peak BLa was measured at 3, 5, and 7 min post-test. Based on data normality, differences in inter-limb step parameters were examined using a paired *t*-test or Wilcoxon signed-rank test. Pearson’s and Kendall’s correlation coefficients evaluated the relationships between BLa and the biomechanical variables (significance level: *a* = 0.05). **Results**: Peak BLa was 16.16 ± 2.61 mmol/L and demonstrated no significant correlation (*p* > 0.05) with sprint time (24.89 ± 1.24 s). Only stride and step frequency during the outer-to-inner leg step was correlated (*p* < 0.05) with BLa. Furthermore, the step initiated by the outer leg had a larger length (*p* < 0.05) compared to the step length generated from the inner leg. **Conclusions**: These findings stem from the different roles between legs while handling the centripetal force requirements during curve sprinting. The outer leg generated a longer step to facilitate curve-to-straight transition, whereas the inner leg functioned primarily to stabilize and steer the body.

## 1. Introduction

The premier sprint event in the Ancient Olympic Games was the stadion (“Στάδιον”), an event nearly equivalent to the modern 200 m dash in terms of distance [1]. Contemporary elite athletes achieve exceptionally high average running speeds (RS), with values up to 11.3 m/s recorded in World Championships [2,3]. It is suggested that success in the 200 m sprint depends heavily on the efficiency of leg mechanics during foot-to-ground contact [4].

In athletics sprints lasting over approximately 9.5 s, the allocation energy derived from phosphocreatine breakdown and glycolysis for muscle contraction depends on the duration, the intensity of the sprinting effort, and the rest intervals between repetitions [5,6]. The depletion of phosphocreatine stores negatively affects RS as the contribution of glycolysis to energy production consequently increases [7]. Given that anaerobic energy stores can be substantially depleted during the 200 m dash [8], it is considered an anaerobic-dominant discipline, with the anaerobic energy system contributing an estimated 70% [8,9] to 80% [10]. This underscores the need to assess on-field biomarkers that provide coaches with essential insights into anaerobic glycolysis, enabling them to design optimum training programs to improve performance [5,11]. A well-established biomarker of energy expenditure in sprinting tasks, and the contribution of anaerobic glycolysis in particular, is blood lactate concentration (BLa) [5,11,12,13]. Lactic acidosis, indicated by increased BLa, is proposed to impair muscle function and drive fatigue in long-sprint events [14]. Previous research suggested that lactic acidosis negatively impacts lower-limb muscle function and sprinting kinematics performance [11], with these fatigue-related changes being linked to a decrease in RS in the final stages of the long-sprint dash [15].

To evaluate sprint technique in track disciplines, the biomechanical factors determining RS need to be identified. In general, RS is determined by stride length (STRL) and stride frequency (STRF), where step length (SL) is the sum of stance distance and flight distance [16]. Step frequency (SF) depends on step time (t_S_), which combines the durations of the ground contact phase (t_C_) and the flight phase (t_FL_) [17]. Both t_C_ and the horizontal distance covered by the body center of mass (BCM) during the stance phase are shaped by body segment configurations at touchdown and take-off, whereas ground reaction forces and air resistance during ground contact determine t_FL_ and the BCM trajectory during the flight phase [16]. Therefore, the examination of the spatiotemporal structure of the sprinting step, alongside the rotational kinematics of body segments and joints, provides a detailed insight into the biomechanics of the sprinting technique related to coordination, power, and stiffness [18].

Stiffness (i.e., the resistance of a body to a change in its length) is essential for sprinting because it affects the aforementioned spatiotemporal parameters, especially SL, SF, t_C_ and t_FL_ [19,20,21]. Evidence suggests that maintaining elevated leg stiffness (K_LEG_) is critical for achieving peak sprinting velocity [22,23]. Furthermore, increased RS has been shown to result in higher peak vertical ground reaction forces (F_Z_), vertical stiffness (K_VERT_) and K_LEG_ [24,25,26], alongside alterations in the kinematic, kinetic, and electromyographic variables that interpret sprinting mechanics [27,28,29].

During a 200 m sprint, RS peaks between the 50 m and 100 m marks before dropping significantly throughout the final 50 m [2,3,15,30,31]. Regarding the home-straight, namely the second 100 m split of the dash, RS typically peaks around the 110–120 m mark [32]. This is due to differences in stride kinematics that occur across different, distinct phases of the same sprinting task [4,11,33,34,35]. As the 200 m sprint progresses, thigh rotational kinematics values decrease [4], negative external work progressively increases, and braking forces increase. This leads to a larger braking-phase duration within the support phase, resulting in greater losses of horizontal velocity during the final meters of the race [35,36]. Furthermore, distinct biomechanical profiles emerge because the 200 m dash is executed on a track featuring both a curved and a straight section [36]. Sprinting on the curve results in greater t_C_ [37,38,39] and, consequently, a lower RS [40]. To maintain their trajectory within the lane, sprinters lean inward toward the center of the curve to generate centripetal force. However, this technical adjustment results in altered lower-limb joint function and increases musculoskeletal loading [41]. Despite these deviations in the sprinting technique in the curve compared to sprinting in the straight, no significant asymmetries are evident in step kinematic parameters (SF, SL, and step velocity) [38,39], since a trade-off between SL and SF seems to maintain inter-limb symmetry regarding step velocity [42]. Nevertheless, the execution of the inner (left) step presents biomechanical disadvantages for maximizing RS, since a longer touchdown distance and an increased thigh separation angle, accompanied by a lower left hip extension velocity, are evident [43]. This occurs because the inner leg must provide stability and steer the body, whereas the outer (right) leg operates more coordinately and efficiently to drive sprinting on the curve [38,42]. Consequently, inter-limb asymmetries have been reported only for the knee joint angle and moment [44].

These differences are particularly evident in the 90–140 m segment of the race, where curved sprinting transitions to straight-line sprinting. Studies have shown that the transition from curve to straight sprinting is characterized by significant decreases in SL [45], even though the 120–140 m segment is the fastest part of the race for many athletes [46]. A recent study found substantial inter-individual variability regarding the length of this transition phase, indicating that sprinters adjust their technique both before and after the geometrical end of the curve [47]. Thus, further research is required to clarify the biomechanical factors of the curve-to-straight transition and its relationship with sprinting technique.

Curve sprinting in the 200 m dash imposes distinct biomechanical requirements under strenuous anaerobic conditions. However, to the best of our knowledge, the available literature lacks substantial evidence regarding the relationship between anaerobic biomarkers and sprinting biomechanics during the transition from curved to straightway sprinting in track and field sprinters. Therefore, the purpose of the present study was to examine the relationship of peak post-test BLa with step kinematic asymmetry and stance-phase biomechanics during the transition from curve to straight-line sprinting in the 200 m dash. Specifically, the relationship of BLa with step kinematic asymmetry, sprinting biomechanical parameters, and spring–mass model characteristics (SMM) was examined. It was hypothesized that BLa would be significantly related to step kinematic asymmetry and the biomechanics of the first stance phase immediately after the junction of the curve and the straightway in the 200 m dash. The information derived from this study will assist coaches in designing more effective training programs that integrate metabolic fatigue with sprints focusing on the critical sprinting biomechanics during the curve-to-straight transition.

## 2. Materials and Methods

### 2.1. Design of the Study

**Figure 1:**
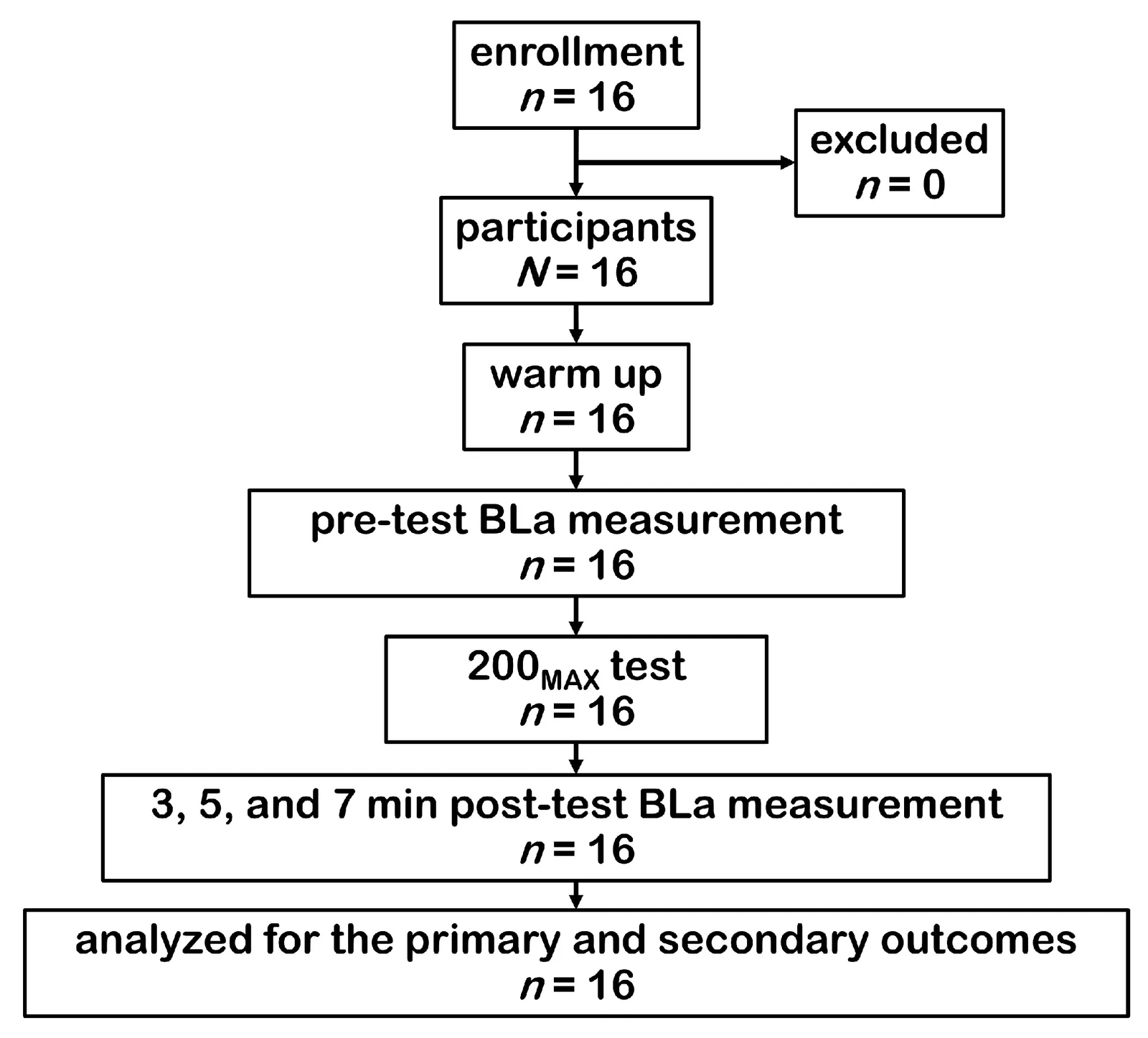
Flowchart of the design and the phases of the cross-sectional study (enrollment, measures, and data analysis). BLa: blood lactate concentration; 200_MAX_: 200 m maximal sprinting test.

To conduct this exploratory cross-sectional study (registered on the AsCollected platform under ID #2280: ‘Sprint biomechanics in the 200 m dash’; https://ascollected.org/5FB_SE5, accessed on 16 August 2026, Supplementary Materials), a cohort of trained young male sprinters from the metropolitan area of Thessaloniki, Greece, was recruited. Participants performed a 200 m maximal sprinting test (200_MAX_), during which pairs of photocells measured their performance. Biomechanical parameters were captured using a video-analysis method, and post-test BLa data was collected after the completion of the 200_MAX_. The dataset was analyzed to test the hypothesis of the study (Figure 1).

### 2.2. Participants

Adult male club- and national-level sprinters trained in the metropolitan area of Thessaloniki, Greece, were recruited to participate in this study. Due to the exploratory nature of the investigation, an a priori sample size calculation was not performed. Before the study commenced, participants were briefed on the experimental procedures and risks, and they provided signed informed consent. The study protocol was approved by the Institutional Research Ethics Committee (Approval Number: 268/2025) and conducted in accordance with the Institutional Research Committee’s guidelines for the ethical treatment of human subjects.

#### 2.2.1. Inclusion Criteria

To be eligible for inclusion, participants were required to be in good health, have a record of competing in the 200 m dash at athletics events, and present no visible or self-reported musculoskeletal injuries or physical limitations.

#### 2.2.2. Exclusion Criteria

Participants were excluded if they attended fewer than five training sessions per week, had sustained an injury within three months before the experimental testing, or had participated in strenuous lower-extremity exercise within 72 h before the tests.

### 2.3. Experimental Procedure

Each participant performed the 200_MAX_ on a certified 400 m outdoor track featuring a World Athletics-approved rubber surface. Before testing, participants completed a standardized 40 min warm-up consisting of 8–10 min of low-intensity running, 10–12 min of lower-limb stretching, neuromuscular coordination drills (i.e., skipping and heel-to-butt kicks), and three 80 m progressive acceleration sprints. Testing was conducted during late spring at the end of the pre-competitive season, between 12:00 and 14:00 h, under ambient temperatures of 22–24 °C. To control for lane configuration and environmental variables [48,49,50,51,52,53,54], all 200_MAX_ trials were performed exclusively in lane 4 (one of the middle lanes). Participants wore their custom competition kit and spikes when performing the experimental sprint test.

#### 2.3.1. Anthropometric Variables and Sporting Profile

Body height and mass were measured using a Seca 220 stadiometer (Seca Deutschland, Hamburg, Germany) and a Delmac PS400L scale (Delmac Instruments S.A., Athens, Greece), respectively. Body height was measured with the participant standing barefoot, facing away from the wall-mounted stadiometer, and keeping their heels together at a 45 deg angle. Participants were instructed to stand as tall as possible and to keep their head at Frankfort horizontal plane. Then, the horizontal lever was lowered to the vertex of the skull and body height was recorded to the nearest 0.001 m. Body mass was measured with participants wearing minimal clothing, without socks or accessories, and the same body posture as described above. After taking a deep breath, body mass was recorded to the nearest 0.1 kg. The body mass index (BMI) was calculated by dividing body mass by height. The sporting profile data, i.e., years of training, training sessions/hours per week, personal bests and level of competition, was acquired using a typical checklist.

#### 2.3.2. Performance

Average RS was calculated as the ratio of distance to time for each 100 m interval, as well as across the entire 200 m distance. Sprint times were recorded electronically using three pairs of photocells (HL 2-34, TAG Heuer Professional Timing, La Chaux-de-Fonds, Switzerland). The corresponding reflectors were mounted on tripods at a height of 1.25 m and positioned at 100 m intervals (0 m/start line, 100 m, and 200 m/finish line). The photocells were connected to an electronic chronometer master console with millisecond accuracy (ARES 21, Omega, Geneva, Switzerland) which was paired with a data printer (Chronoprinter 503, TAG Heuer Professional Timing, La Chaux-de-Fonds, Switzerland). To eliminate the confounding effect of reaction time on sprint performance, participants initiated the test using a standing start technique positioned 1 m behind the first pair of photocells [11].

#### 2.3.3. Kinematics

Lane 4 was also selected to video-record the participants as they transitioned from the curve into the straight section of the 200_MAX_. Three consecutive ground support phases were recorded at approximately the 115 ± 5 m mark, with the objective of capturing the middle support phase as the first step at the straightway.

The steps were recorded from the left side of the participants using a stationary digital video camera (JVC GR-DVL 9600EG, Victor Co., Yokohama, Japan) operating at a sampling frequency of 100 fps. The camera was mounted on a fixed tripod at a height of 1.2 m, positioned 20 m away from the inside lane, with its optical axis aligned perpendicular to the direction of the straightway. The camera lens was zoomed to capture a 9 m wide field of view.

To perform a two-dimensional direct linear transformation (2D-DLT) kinematic analysis [55], a 2.5 × 2.5 m calibration frame with 16 control markers was placed within the field of view along the center of the lane and perpendicular to the camera’s optical axis. The *X*-axis represented the horizontal direction of running, whereas the *Y*-axis represented the vertical axis. Eighteen anatomical landmarks (vertex of the head, and bilaterally the tip of the toe, ankle, knee, hip, shoulder, elbow, wrist, and fingertips), along with selected reference points in the recorded field of view, were manually digitized in each video frame. Segmental inertia properties suggested by Plagenhoef [56] were used for the link-segment model to calculate the BCM kinematic parameters.

Data digitization, smoothing, and subsequent analyses were performed using the A.P.A.S. v.14.1.05 software (Ariel Dynamics Inc., Trabuco Canyon, CA, USA). A second-order low-pass Butterworth digital filter with a cut-off frequency of 6 Hz was selected for smoothing after visual inspection of the data. To assess the reliability of the manual digitization process, the accuracy of the 2D reconstruction was determined by root mean square (RMS) error after randomly re-digitizing 10% of the captured video frames.

The extracted XY anatomical coordinates were used to calculate the following biomechanical parameters:

- SF: 1/t_S_.
- SL: horizontal distance between consecutive foot touchdowns.
- STRL: the addition of two consecutive SLs.
- STRF: the division of 1 to the addition of t_S_ for two consecutive steps.
- Touchdown distance: horizontal distance from support toes to BCM projection at touchdown.
- Take-off distance: horizontal distance from support toes to BCM projection at take-off.
- Horizontal BCM touchdown and take-off velocity.
- Vertical BCM touchdown and take-off velocity.
- Step take-off angle: arctangent of vertical to horizontal BCM velocity at take-off.
- Horizontal ankle touchdown velocity: horizontal ankle velocity at touchdown.
- BCM height: vertical BCM position at touchdown, maximum lowering, and take-off.
- Knee angle: the thigh–shank angle at touchdown, maximum flexion, and take-off.
- Minimum swing knee angle: minimum thigh–shank angle of the swing leg during the support phase.
- Ankle joint angle: shank–foot angle at touchdown and take-off.
- Ankle joint range of motion: the difference in ankle joint angle between touchdown and take-off.
- Knee angular velocity: maximum angular velocity of the knee during the support phase.
- Ankle angular velocity: angular velocity of the ankle at touchdown.
- Ankle relative horizontal velocity: the difference between the horizontal BCM and the horizontal ankle velocity at touchdown.
- Thigh inclination: horizontal-to-thigh angle of the swing leg at take-off in the sagittal plane.
- Leg inclination: horizontal-to-hip/ankle line angle of the support leg at touchdown and take-off in the sagittal plane.

The BCM kinematics and the joint angular rotation kinematics corresponding to the lower extremity executing the second step of the three (i.e., the first step in the straightway) captured with the video-recording were included in the statistical analysis. Inter-limb asymmetry for SL and SF between the inner (left) and the outer (right) leg was quantified using absolute symmetry angle values, which were calculated according to the method proposed by Exell et al. [57].

#### 2.3.4. Spring–Mass Model Parameters

SMM variables were calculated using the method presented by Morin et al. [58]. The accuracy of the method is reported to be ±3.2%, ±2.3% and ±2.5% for F_Z_, K_VERT_ and K_LEG_, respectively.

#### 2.3.5. Blood Lactate

To evaluate the anaerobic capacity of the participants, BLa was also measured. To determine blood lactate concentration, BLa, capillary blood samples were collected from a fingertip at baseline and at 3, 5, and 7 min post-test. Samples were immediately diluted in a 10-fold volume of 0.3 mol/L perchloric acid and stored at –20 °C prior to analysis. On the day of analysis, samples were thawed and centrifuged at 1500 *g* for 5 min. Lactate concentration was then quantified in the supernatant using an enzymatic photometric method (no. 826-UV, Sigma Diagnostics, St. Louis, MO, USA). The coefficient of variation for lactate was 3% [11]. The highest value from these measurements was selected for further analysis.

### 2.4. Statistical Analysis

Statistical procedures were accomplished using IBM SPSS Statistics v.29 (ΙBM Corp., Armonk, NY, USA). The level of statistical significance was set at *a* = 0.05.

Depending on the results of the Shapiro–Wilk test, the relationship among the examined variables was examined by Pearson’s and Kendall’s rank correlation. Values within the range of 0.00–0.09, 0.10–0.39, 0.40–0.69, 0.70–0.89, and 0.90–1.00 for Pearson’s *r_P_* correlation coefficient and 0.00–0.05, 0.06–0.25, 0.26–0.48, 0.49–0.70, and 0.71–1.00 for Kendall’s *τ_b_* correlation coefficient were interpreted as negligible, weak, moderate, strong, and very strong relationships, respectively [59,60]. To account for the small sample size (*N* = 16) relative to the number of correlation analyses performed, *p*-values were adjusted using the Benjamini–Hochberg false discovery rate (FDR) procedure. Given the exploratory nature of the data analysis, *q*-values were extracted using the online multipletesting.com application [61] with a target FDR threshold set at 10%.

Differences between the inner and outer leg for SL were examined with a paired-samples *t*-test, whereas possible differences between the inner and outer leg for SF were examined using the Wilcoxon signed-rank test. For the former, the effect size was evaluated with Cohen’s *d* (*d* < 0.2: small; 0.2 ≤ *d* < 0.8: medium; *d* ≥ 0.8: large). For the latter, the *r* effect size coefficient was used to interpret the magnitude of the differences (*r* < 0.1: small; 0.3 ≤ *r* < 0.5: medium; *r* ≥ 0.5: large) [62].

## 3. Results

Sixteen adult male club- and national-level 200 m sprinters volunteered to participate in the study. Table 1 presents descriptive information about the participants and their sporting profile.

Results indicated that maximum post-test BLa was 16.16 ± 2.61 mmol/L, and it was not correlated (*p* > 0.05) with performance in 200_MAX_ and the separate 100 m splits nor with RS in any of the examined distances (Table 2).

**Figure 2:**
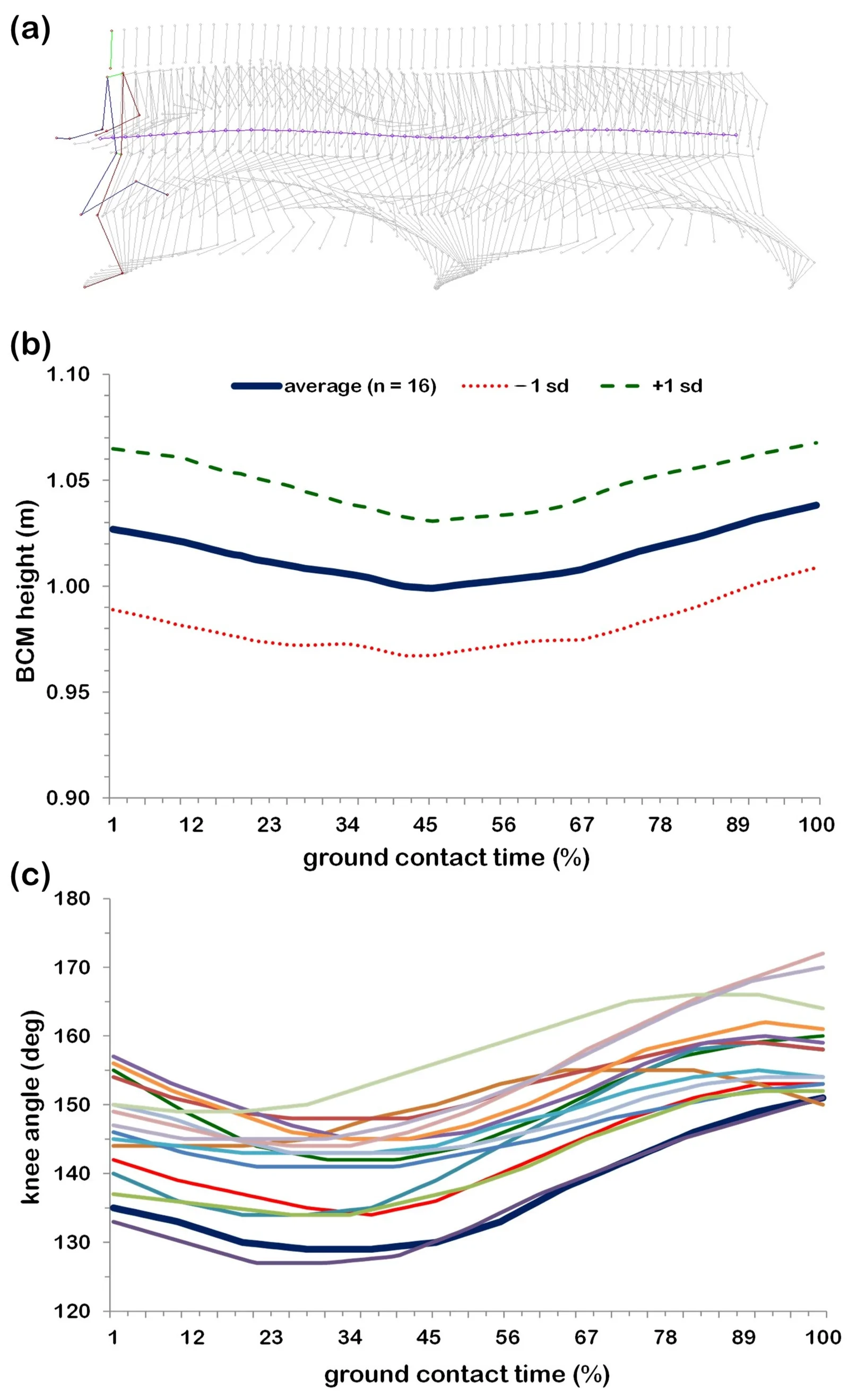
Graphical depiction of the outcomes of the 2D-DLT analysis. (**a**) Representative stick figure (top panel). (**b**) Ensemble average (*n* = 16) time history curve for the body center of mass (BCM) during the ground contact phase (middle panel). (**c**) Individual (*n* = 16) time curves for the knee angle during the ground contact phase (bottom panel).

The 2D reconstruction revealed RMS errors of 7.7 mm and 7.5 mm for the X- and Y-axes, respectively. Typical outcomes of the 2D-DLT analysis are depicted in Figure 2.

The results of the linear kinematic parameters examined and their correlation with maximum post-test BLa are presented in Table 3. It was found that SF was positively moderately correlated with BLa (*p* < 0.05, *q* = 0.020 after FDR adjustment). No other significant correlation was revealed (*p* > 0.05).

In nine participants, the analyzed support leg was the outer leg, while in seven participants, it was the inner leg. No significant correlations (*p* > 0.05) were found between BLa and the examined joint rotational kinematic and SMM parameters (Table 4).

The association analysis between the step kinematic parameters and BLa revealed that SF for the step initiated from the outer to the inner leg was positively moderately correlated with BLa (*p* < 0.05, *q* = 0.020 after FDR adjustment; Table 5). In addition, this step was found to be significantly larger than the step initiated from the inner leg (*t*_1,15_ = 2.880, *p* = 0.011, *d* = 0.72, medium effect size). Regarding SF, no differences (*p* > 0.05) were found between the two legs (*Z* = 0.943, *p* = 0.345, *r* = 0.17, medium effect size).

**Figure 3:**
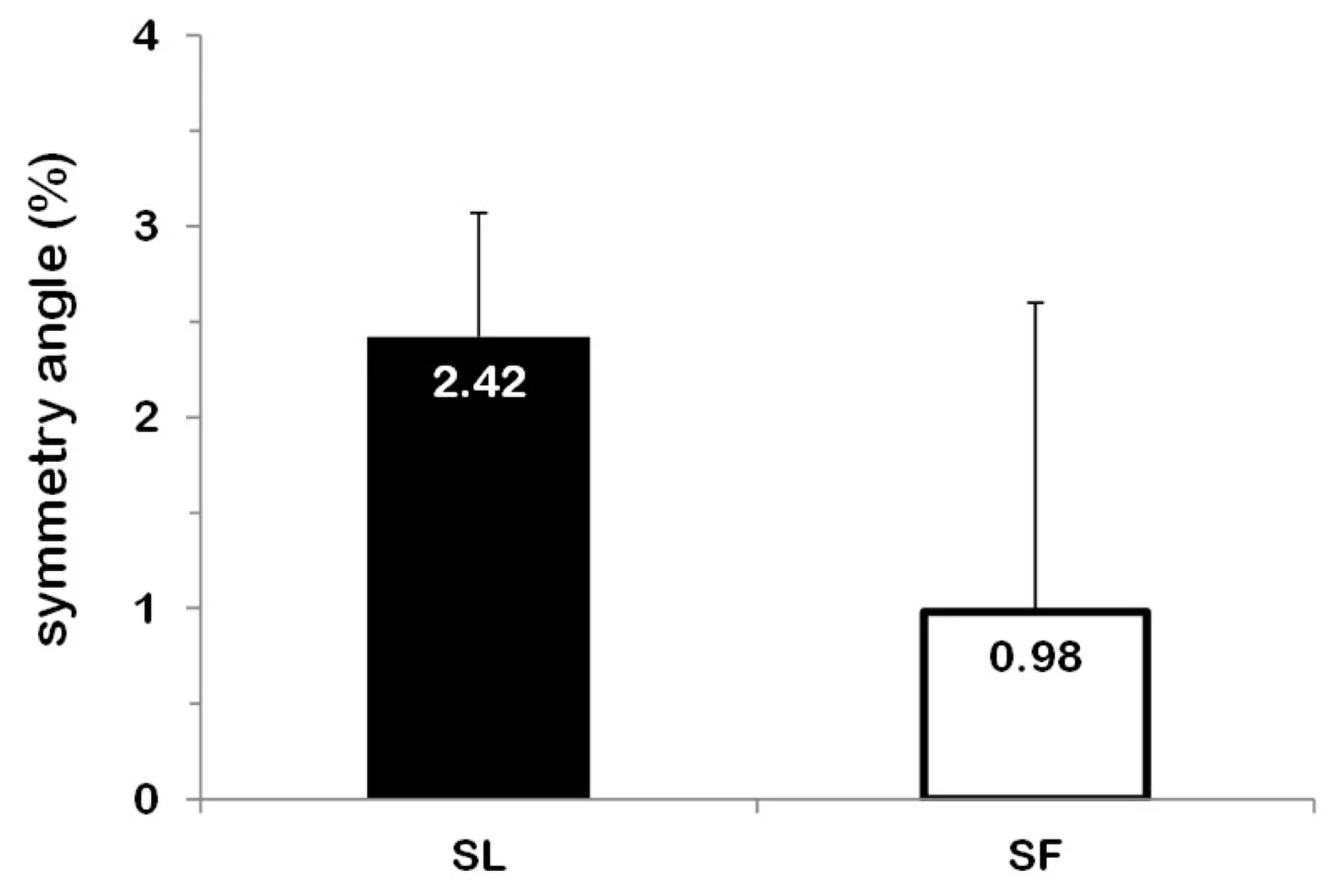
Descriptive results for the step length (SL) and step frequency (SF) symmetry angles (*n* = 16).

Finally, the results for the asymmetry in the examined step kinematic parameters are presented in Figure 3. The symmetry angles for SL (*r* = 0.265, *p* = 0.322) and SF (*v_b_* = 0.053, *p* = 0.794) were not correlated (*p* > 0.05) with BLa.

## 4. Discussion

The initial hypothesis was partially supported, as BLa was related neither to the kinematic and SMM parameters of the first ground support phase on the 200 m straightway, nor to overall step parameter asymmetry. Specifically, the biomechanical parameters of the support phase were independent of BLa; rather, BLa correlated with STRF and SF of the step initiated by the outer leg.

Anaerobic energy is derived from two sources: alactic and lactic (glycolytic) metabolism. The latter is typically evaluated using BLa accumulation during exercise as an indicator, despite the confounding effects of muscle fiber distribution and body composition [63]. Post-test BLa in the present study was higher than values previously reported for male 200 m sprinters [8,12,63]. Nevertheless, these values agree with those reported for male students with previous experience as track runners [11]. The higher values recorded in the present study may be attributed to the slower performance times of the participants compared to past studies [8,12,42]. As performance time increases, the exercise duration approaches the time frame required to attain the maximum rate of BLa accumulation, thereby yielding higher expected BLa values due to the slightly greater anaerobic contribution when the duration of the task is approaching 30 s [7,64]. However, sprinters of varying competitive levels exhibit differences in BLa, with higher values indicating greater glycolytic contribution in higher-level athletes [5]. During periods of high anaerobic energy expenditure, sprinters utilize complex neuromuscular adjustments to maintain leg rigidity [24,25,26]. This is supported by past findings that sprinters with increased K_LEG_ possess an advantage during curved sprinting [42]. The present study revealed no evidence of a relationship between BLa and joint biomechanics or stiffness during the curve-to-straight transition phase. This suggests that the ability to maintain macro-level sprinting biomechanics when in maximal effort was independent of the metabolic fatigue induced by the 200_MAX_ test.

The significant correlation between SF and maximum post-test BLa suggests that elevated neuromuscular activity drives greater activation of anaerobic energy pathways since rapid anaerobic energy release is typically stimulated by muscle activity during maximal sprinting [64]. Elevated lactate levels indicate intense anaerobic glycolytic activity and consequent metabolic acidosis, which can ultimately impair muscle force application during ground contact [12,14]. Although electromyographic activity does not differ between straight and curved sprinting, findings suggest higher activation of the medial head of the gastrocnemius of the inner leg [65]. Notably, increased BLa has been associated with less inter-limb asymmetry in muscle activation among female sprinters, indicating that sprinters exhibit enhanced neuromuscular coordination under strenuous conditions [66]. Thus, training regimes for 200 m sprinters should focus on high horizontal force application, short ground contact, and delayed propulsive-force decline, all while managing the metabolic cost of a predominantly anaerobic effort that still draws a meaningful aerobic contribution [8,36].

While overall SL asymmetry was not extensive, a significant inter-limb discrepancy emerged, contradicting past findings for the curved sprinting phase [42,45,67], as the outer-to-inner SL exceeded the inner-to-outer SL. This asymmetrical mechanical behavior arises from the centripetal force requirements of curved running, which dictate distinct functional roles for each limb and alter lower-extremity joint loading to accommodate the body’s inward lean [32,68]. The body’s inward lean is associated with the magnitude of the inward impulse generated by the inner leg, which is further related to rapid knee and hip extension velocities at take-off [69]. Conversely, the step initiated by the outer leg is characterized by a large vertical impulse and reduced t_C_ resulting from decreased hip and knee flexion at touchdown [69]. These technique elements underscore the inter-limb differences in the role of each lower extremity during curved sprinting; specifically, the step initiated by the outer leg appears to optimize sagittal-plane kinetics, whereas the step initiated by the inner leg acts to control the lateral kinetics and kinematics while optimizing rapid leg extension [69].

The present findings for the curve-to-straightway transition phase suggest that the inter-limb differences emerging during curve sprinting persist into this subsequent phase of the 200 m dash. Specifically, a longer SL is generated by the outer leg to drive forward propulsion, whereas the inner leg functions primarily to stabilize and steer the body. This stabilizing role limits the explosive capacity of the inner leg, which must also withstand additional mechanical loading [70]. Conversely, the outer leg generates forward propulsion more efficiently [42]. This underscores that the biomechanics of the curved-to-straight transition should not be viewed merely as an intermediate blend of curved and straight-line sprinting, but rather as a distinct biomechanical phenomenon in its own right.

Several limitations should be considered when generalizing the findings of this study. First, regarding the sample size, the sample size was small (*N* = 16). The results of a power analysis [71] for an effect size greater than 0.5, a probability error of 0.05, and a power of 0.80 revealed that a sample size of 26 participants should have been considered sufficient. However, Kendall’s *v_b_* correlation coefficients for the relationship between BLa and SF achieved ~89.6% statistical power, indicating an exceptionally strong relationship [72]. Additionally, the participants were exclusively men; therefore, caution is warranted when extrapolating these findings to women sprinters due to sex-based differences in muscle volume and other morphological variables that typically result in lower sprint parameters compared to men [73]. Another limitation involves using post-exercise BLa as an indirect bioenergetic marker. However, it remains a widely accepted biomarker for assessing glycolytic contribution in maximum-intensity training regimes and serves as an indicator of an individual’s “metabolic profile” [5]. Furthermore, no tests were conducted to examine potential inter-limb asymmetries in the neuromuscular function of the lower extremities, despite evidence that neuromuscular fatigue in sprinters can be detected using standard vertical jump tests [74]. Regarding the biomechanical analysis, sprinting technique adjustments during the transition from the curve to the straightway range from a minimum of 7.39 m to a maximum of 37.90 m [47]. Consequently, the 2D-DLT analysis used in this study may not have captured potential movement adjustments occurring outside the camera plane, unlike typical biomechanical studies that utilized three-dimensional kinematic analysis [37,38,47]. Similarly, direct ground reaction force data were not collected to measure SMM variables, despite the documented statistical similarity between the method by Morin et al. [58] used in the present study and typical kinetic–kinematic analysis [75]. Electromyographic data could also have provided deeper insight into muscle activation differences between the inner and outer legs [76]. Finally, the pacing strategy for the two 100 m splits in the 200_MAX_ was not controlled, even though pacing in the 200 m dash is suggested to influence overall performance [15].

Future research should be conducted to examine the above-mentioned adjustments over a broader segment of the 200 m dash, spanning both the pre- and post-geometric termination of the curve. Furthermore, longitudinal or cross-sectional studies regarding the developmental trajectory of these adjustments, alongside interventions assessing the effectiveness of training modalities aimed at improving coordination and stiffness, would elucidate the underlying mechanisms. Mechanistically, the clinical and practical applicability of this research primarily lies in the optimization of specialized training regimes for track and field sprinters. The outcomes of the study indicate that metabolic fatigue is independent of macro-level joint biomechanics during the curve-to-straight transition. Crucially, the inter-limb asymmetry revealed for SL underscores the differences in physical loading during the curve-to-straight transition. This highlights the necessity for limb-specific training strategies that simultaneously challenge metabolic capability alongside the regulation of the unilateral loading patterns required to effectively negotiate the curve–straightway junction in long-sprint disciplines.

## 5. Conclusions

Peak post-test BLa did not correlate with joint rotational kinematics, SMM stiffness, or overall step asymmetry angles of the first stance phase immediately after the junction of the curve and the straightway in the 200 m dash. However, an explicit inter-limb step length asymmetry was revealed for this step. These findings demonstrate that the curve-to-straight transition phase is a distinct phenomenon requiring targeted training regimens focusing on unilateral loading to safely handle the distinct loading patterns of each leg. Therefore, pacing and conditioning programs must integrate metabolic fatigue handling with curve-to-straight transition drills. Nevertheless, further investigation to account for developmental trajectories and pacing variables is needed.

## Figures and Tables

**Table 1:** Descriptive information about the participants and their sporting profile (*n* = 16).

Parameter	Mean ± SD	Range	95% CI
age (years)	21.30 ± 1.00	20.30–22.30	20.77–21.83
body height (m)	1.81 ± 0.05	1.74–1.90	1.78–1.83
body mass	74.91 ± 6.50	64.00–86.10	71.73–78.10
BMI (kg/m^2^)	22.81 ± 1.21	21.14–25.25	22.22–23.40
national:club level (%)	50%:50%		
training sessions per week	5.94 ± 1.06	5.00–8.00	5.42–6.46
duration of training per week (h)	12.31 ± 2.12	10.00–16.00	11.27–13.35
200 m personal best (s)	24.30 ± 1.21	22.44–26.25	23.71–24.89
200 m World Athletics score (points)	643.18 ± 136.20	434.00–866.00	576.44–709.92

SD: standard deviation; CI: confidence interval; BMI: body mass index.

**Table 2:** Performance parameters and their correlation with maximum post-test blood lactate concentration (*n* = 16).

Parameter	Mean ± SD	95% CI	Correlation Coefficient	*p*
200 m performance (s)	24.89 ± 1.24	24.05–25.72	−0.176 ^¶^	0.605
200 m average speed (m/s)	8.06 ± 0.40	7.79–8.32	0.176 ^¶^	0.606
0–100 m performance (s)	12.63 ± 0.64	12.20–13.07	−0.099 ^¶^	0.772
0–100 m average speed (m/s)	7.93 ± 0.40	7.66–8.21	0.100 ^¶^	0.771
100–200 m performance (s)	12.56 ± 0.81	12.02–13.10	−0.054 ^¶^	0.876
100–200 m average speed (m/s)	8.00 ± 0.51	7.65–8.34	0.062 ^¶^	0.876

SD: standard deviation; CI: confidence interval; ^¶^: value of Pearson’s *r* correlation coefficient; *p*: significance level.

**Table 3:** Linear kinematic parameters and their correlation with maximum post-test blood lactate concentration (*n* = 16).

Parameter	Mean ± SD	95% CI	Correlation Coefficient	*p*
Stride length (m)	4.80 ± 0.72	4.42–5.18	0.041 ^¶^	0.879
Stride length (% body height)	241.14 ± 19.55	228.04–254.31	−0.007 ^¶^	0.983
Stride frequency (Hz)	1.99 ± 0.12	1.91–2.07	0.567 ^#^*	0.022
Step time (ms)	256 ± 16	246–270	−0.396 ^¶^	0.129
Contact time (ms)	114 ± 12	109–125	−0.329 ^¶^	0.214
Contact time (% step time)	45.78 ± 4.21	42.95–48.61	−0.101 ^¶^	0.701
Flight time (ms)	141 ± 15	131–151	−0.318 ^¶^	0.231
Horizontal BCM touchdown velocity (m/s)	8.38 ± 1.06	7.66–9.10	0.152 ^#^	0.574
Vertical BCM touchdown velocity (m/s)	−0.56 ± 0.12	−0.64–−0.48	−0.311 ^¶^	0.242
Horizontal BCM take-off velocity (m/s)	8.57 ± 1.01	7.89–9.25	0.142 ^¶^	0.601
Vertical BCM take-off velocity (m/s)	0.65 ± 0.15	0.55–0.75	0.067 ^¶^	0.807
Angle of projection (°)	4.36 ± 0.93	3.73–4.98	−0.017 ^¶^	0.950
Horizontal BCM touchdown distance (m)	0.42 ± 0.11	0.34–0.50	0.065 ^#^	0.812
Horizontal BCM take-off distance (m)	0.59 ± 0.06	0.55–0.63	−0.069 ^#^	0.798
Horizontal BCM distance at support (m)	0.98 ± 0.10	0.91–1.05	−0.068 ^¶^	0.804
Horizontal BCM distance at flight (m)	1.24 ± 0.23	1.08–1.39	0.008 ^¶^	0.976
BCM height at touchdown (m)	1.02 ± 0.04	0.99–1.05	−0.417 ^¶^	0.108
Minimum BCM height during support phase (m)	0.99 ± 0.04	0.96–1.02	−0.493 ^¶^	0.052
Lowering of BCM during support phase (m)	−0.03 ± 0.02	−0.04–−0.02	−0.063 ^¶^	0.815
BCM height at take-off (m)	1.04 ± 0.03	1.01–1.06	−0.382 ^¶^	0.144

*: *p* < 0.05; SD: standard deviation; CI: confidence interval; ^¶^: value of Pearson’s *r_P_* correlation coefficient; ^#^: value of Kendall’s *τ_b_* correlation coefficient; *p*: significance level.

**Table 4:** Joint angular kinematic parameters and spring–mass characteristics and their correlation with maximum post-test blood lactate concentration (*n* = 16).

Parameter	Mean ± SD	95% CI	Correlation Coefficient	*p*
Knee angle at touchdown (°)	148.82 ± 7.07	144.07–153.57	0.207 ^¶^	0.442
Minimum knee angle during support phase (°)	142.45 ± 6.39	138.16 ± 146.75	0.084 ^¶^	0.757
Knee angle flexion (°)	−6.36 ± 3.36	−8.62–−4.11	−0.290 ^¶^	0.275
Knee angle at take-off (°)	158.82 ± 6.81	154.24–163.39	−0.144 ^¶^	0.596
Maximum support leg’s knee angular velocity during support phase (rad/s)	16.90 ± 2.55	15.18–18.61	0.393 ^¶^	0.132
Ankle angle at touchdown (°)	101.73 ± 7.56	96.65–106.81	0.249 ^¶^	0.353
Ankle relative horizontal velocity at touchdown (m/s)	−7.06 ± 0.72	−7.54–−5.58	−0.227 ^¶^	0.399
Support leg’s ankle angular velocity at touchdown (rad/s)	−5.66 ± 1.56	−6.71–−4.61	−0.314 ^¶^	0.236
Ankle angle at take-off (°)	123.73 ± 9.08	117.63–129.83	−0.150 ^#^	0.580
Ankle joint range of motion (°)	22.00 ± 8.57	16.24–27.76	−0.340 ^¶^	0.198
Minimum swing leg’s knee angle during support phase (°)	34.55 ± 7.85	29.27–39.82	0.048 ^¶^	0.860
Maximum swing leg’s knee angular velocity during support phase (rad/s)	18.39 ± 2.08	16.99–19.79	0.422 ^¶^	0.103
Support leg inclination at touchdown (°)	74.50 ± 5.35	70.91–78.09	−0.054 ^¶^	0.841
Support leg inclination at take-off (°)	50.15 ± 5.03	46.77–53.53	0.095 ^¶^	0.725
Swing leg’s thigh inclination at take-off (°)	21.00 ± 5.55	17.27–24.73	−0.170 ^¶^	0.529
Peak vertical ground reaction force (kN)	2.55 ± 0.29	2.36–2.75	−0.377 ^¶^	0.254
Vertical stiffness (N/m)	125.21 ± 79.10	72.07–178.35	−0.146 ^#^	0.669
Leg stiffness (kN/m)	16.26 ± 3.29	14.05–18.47	−0.255 ^¶^	0.450

SD: standard deviation; CI: confidence interval; ^¶^: value of Pearson’s *r_P_* correlation coefficient; ^#^: value of Kendall’s *τ_b_* correlation coefficient; *p*: significance level.

**Table 5:** Step kinematic parameters and their correlation with maximum post-test blood lactate concentration (*n* = 16).

Parameter	Mean ± SD	95% CI	Correlation Coefficient	*p*
outer-to-inner leg step length (m)	2.42 ± 0.36	2.23–2.62	0.026 ^¶^	0.924
inner-to-outer leg step length (m)	2.37 ± 0.35	2.19–2.56	0.056 ^¶^	0.837
outer-to-inner leg step frequency (Hz)	3.95 ± 0.21	3.84–4.06	0.528 ^#^*	0.008
inner-to-outer leg step frequency (Hz)	4.00 ± 0.29	3.85–4.15	0.324 ^#^	0.101

*: *p* < 0.05; SD: standard deviation; CI: confidence interval; ^¶^: value of Pearson’s *r* correlation coefficient; ^#^: value of Kendall’s *v_b_* correlation coefficient; *p*: significance level.

## Data Availability

The data presented in this study are available on request from the corresponding author due to ethical reasons.

## Supplementary Materials

The following supporting information can be downloaded at: https://www.mdpi.com/article/10.3390/jfmk11030335/s1.

## Abbreviations

The following abbreviations are used in this manuscript:

200_MAX_	200 m maximal sprinting test
2D-DLT	two-dimensional direct linear transformation
BCM	body center of mass
BMI	body mass index
BLa	peak blood lactate concentration
CI	confidence interval
FDR	false discovery rate
F_Z_	vertical ground reaction forces
K_LEG_	leg stiffness
K_VERT_	vertical stiffness
RMS	root mean square
RS	average running speed
SD	standard deviation
SF	step frequency
SL	step length
SMM	spring–mass model
STRF	stride frequency
STRL	stride length
t_C_	ground contact time
t_FL_	flight time
t_S_	step time
*X*-axis	horizontal axis
*Y*-axis	vertical axis
