# Supplementary material for: Blood Lactate’s Relationship with Step Kinematic Asymmetry, Stance-Phase Biomechanics, and Spring–Mass Model Variables at the Transition from Curve to Straight Sprinting in the 200 m Dash: A Cross-Sectional Study
Source: J Funct Morphol Kinesiol. 2026 Aug 26;11(3):335. doi: 10.3390/jfmk11030335 (PMC13608454; doi:10.3390/jfmk11030335)
Supplement: Supplementary file 1 [file jfmk-11-00335-s001.zip › jfmk-4463087-R1_supplement_EthicalApproval_240826-S.pdf]

ARISTOTLE UNIVERSITY OF THESSALONIKI	**FACULTY OF PHYSICAL EDUCATION AND SPORT SCIENCES**<br>**SCHOOL OF PHYSICAL EDUCATION AND SPORT SCIENCE AT THESSALONIKI**<br>**RESEARCH ETHICS COMMITTEE**<br><br>Info: Ms Eleanna Manoussaridou<br>Phone: +30 2310 99 1602<br>Email: ethicscommittee@phed.auth.gr<br>1st floor, Glass building, Thermi campus

Thessaloniki, July 17, 2026

Approval number 353/2026

APPROVAL OF STUDY DESIGN

The Research Ethics Committee of the School of Physical Education and Sport Science at Thessaloniki approves the research design entitled "Blood Lactate Is Not Related to Step Kinematic Asymmetry and Stance Phase Biomechanics at the Transition From Curve to Strait Sprinting in the 200-m Dash in Athletics", as submitted retrospectively by Dr Vasileios Panoutsakopoulos, for the following reasons:

• no evidence indicates that the research posed risks to the participants,
• the Committee is not aware of any breach of participant anonymity or data confidentiality,
• the submitted documents indicate that the criteria of good scientific practice were followed, in accordance with the Regulation of Principles and Operation of the Research Ethics Committee of Aristotle University of Thessaloniki and the applicable legislation.

On behalf of the Committee

Vassilis Barkoukis

Professor

Director of Ethics Committee
